# Ten-year outcomes of coronary artery bypass grafting versus percutaneous coronary intervention in patients with three-vessel disease and heart failure

**DOI:** 10.1016/j.ahjo.2025.100659

**Published:** 2025-10-30

**Authors:** Jimmy Kang, Ryaan El-Andari, Nicholas Fialka, Yongzhe Hong, Michael S. McMurtry, Jeevan Nagendran, Jayan Nagendran

**Affiliations:** a Division of Cardiac Surgery, Department of Surgery, Canada; b Department of Medicine, University of Alberta, Edmonton, Alberta, Canada

**Keywords:** Coronary artery bypass grafting, Percutaneous coronary intervention, Coronary artery disease, Coronary revascularization, Left ventricular dysfunction

## Abstract

**Objective:**

The optimal revascularization strategy for patients with three-vessel coronary artery disease (3VD) and heart failure (HF) remains uncertain due to the absence of randomized trials directly comparing coronary artery bypass grafting (CABG) and percutaneous coronary intervention (PCI). With few observational studies providing long-term follow-up, clinical equipoise persists. We therefore evaluated 10-year outcomes between CABG and PCI in patients with HF and 3VD.

**Methods:**

This retrospective population-based cohort study included adults with 3VD and HF undergoing isolated CABG or PCI in Edmonton, Alberta, Canada (2009–2018). Patients with STEMI, prior CABG, or concomitant procedures were excluded. The primary endpoint was all-cause mortality. Secondary endpoints included readmission for myocardial infarction (MI), stroke, repeat revascularization, and all-cause rehospitalization. Multivariable Cox regression was used to adjust for baseline characteristics.

**Results:**

Of 1774 screened patients, 632 met inclusion criteria (CABG: n = 97; PCI: n = 535). At 10 years, all-cause mortality was significantly lower in the CABG group (62.4 %) compared to PCI (71.8 %) (adjusted hazard ratio [aHR] 0.65, 95 % CI 0.47–0.92; *p* = 0.014). CABG was also associated with markedly lower rates of MI readmission (3.2 % vs. 23.7 %; aHR 0.11, 95 % CI 0.03–0.38; *p* < 0.001) and repeat revascularization (6.4 % vs. 21.6 %; aHR 0.22, 95 % CI 0.09–0.53; *p* = 0.001). Rates of stroke (*p* = 0.757) and all-cause rehospitalization (*p* = 0.157) were not significantly different.

**Conclusions:**

In patients with 3VD and HF, CABG is associated with significantly improved long-term survival, reduced MI readmissions, and fewer repeat revascularizations compared to PCI. These findings reinforce the need for a multidisciplinary Heart Team review to ensure the optimal intervention strategy.

## Highlights

• 632 patients with three vessel coronary disease and heart failure underwent either CABG or PCI
• 10-year mortality was significantly lower in patients undergoing CABG compared to PCI (*p* = 0.015)
• Readmission for myocardial infarction and repeat revascularization also favored CABG
• Rehospitalization and stroke did not differ significantly between groups
• CABG provides significant long term benefits over PCI for patients with multivessel disease and heart failure

## Graphical abstract

**Figure:**
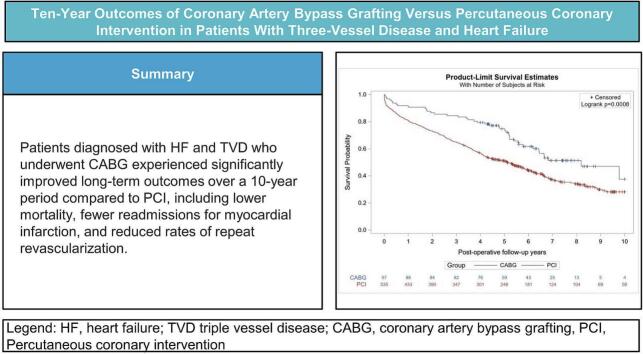

## 1 Introduction

Coronary artery disease (CAD) is the most common cause of heart failure (HF), and the incidence of ventricular dysfunction among patients with CAD is increasing with improved survival after acute myocardial infarction [[1], [2], [3], [4], [5], [6], [7], [8], [9]]. The optimal revascularization strategy in patients with three-vessel disease (3VD) presenting with heart failure remains debated due to underpowered randomized controlled trials and limited long-term studies comparing the outcomes of percutaneous coronary intervention (PCI) versus coronary artery bypass grafting (CABG) [[10], [11], [12], [13], [14], [15]]. While the European guideline lists CABG as a class IIa indication and PCI as a class IIb for those with multivessel disease and HF [16], the North American guideline only lists CABG as a class I indication for this patient population, with no recommendations regarding the PCI revascularization route [16].

Due to the lack of consensus in the optimal revascularization strategy and paucity of adequate evidence, patients with 3VD and HF have received various treatments as per the operator's expertise, co-existing comorbidities, and competing risks. Herein, we compare the long-term outcomes of a real-world cohort of patients with 3VD and a clinical diagnosis of HF undergoing PCI or CABG, with up to 10 years of follow-up.

## 2 Methods

### 2.1 Data source

A population-based cohort was generated from records of patients who underwent angiography in the province of Alberta, Canada. Data were collected in the APPROACH (Alberta Provincial Project for Outcome Assessment in Coronary Heart Disease) database. Data included preoperative and demographic characteristics, intraprocedural details, catheterization results, and postprocedural outcomes. Additional preoperative and postoperative data, including hospitalizations and mortality, were obtained from discharge abstract databases and the Alberta provincial death registry. Ethics approval was granted by the University of Alberta Health Research Ethics Board for study ID Pro00124631.

### 2.2 Study cohort

**Fig. 1:**
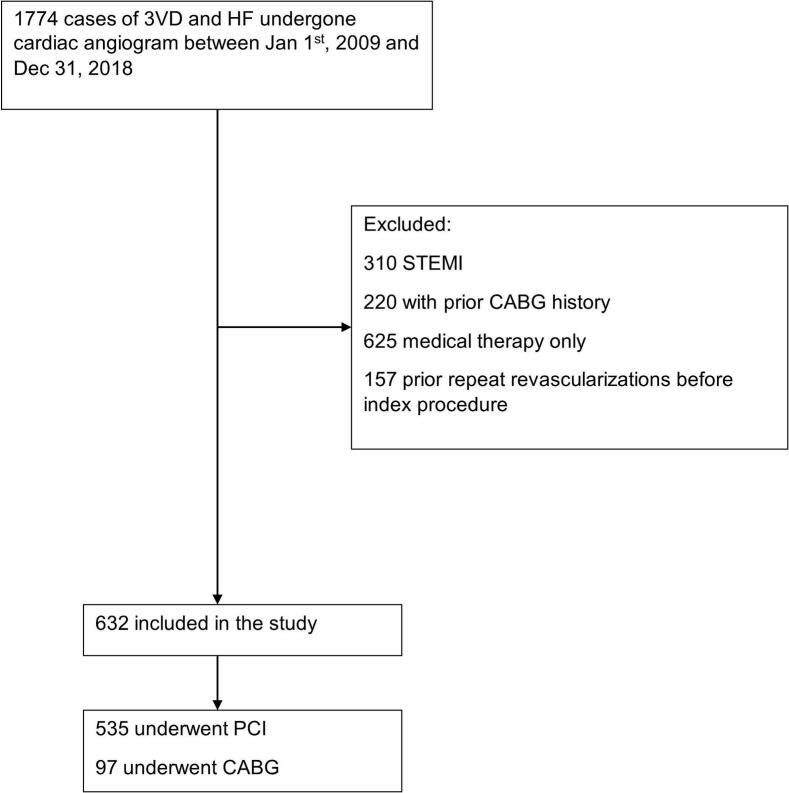
Flow diagram of patients selection.

The inclusion criteria for this study were patients >18 years of age with a clinical diagnosis of heart failure and TVD on coronary angiography between January 1, 2009 and December 31, 2018 (Fig. 1). Clinical diagnosis of heart failure included acute, chronic, systolic, diastolic, left ventricular or bi-ventricular heart failure and the detailed ICD codes used were included in Supplementary Table 1. Patients were required to undergo revascularization with either isolated CABG or PCI. Patients were assigned to their respective groups (CABG vs. PCI) at the time of the first coronary intervention. Exclusion criteria were: (i) ST-elevation myocardial infarction (STEMI) as it is infrequently treated with CABG and would bias against the PCI group, (ii) a history of prior CABG, (iii) patients receiving medical therapy only, and (iv) prior revascularization before index procedure. Patients with a history of CABG prior to the index revascularization were excluded from the analytic cohort; their angiograms appear only in the screened population, and Surgical cases with concomitant valve procedures were excluded before linkage to the angiography cohort and therefore do not appear in the 1774-patient denominator. All comorbidities were defined according to APPROACH forms and data, based on diagnoses assigned by the attending physician at the time of angiography. Lesions with ≥50 % stenosis were defined as significant in APPROACH and multivessel disease was defined as the presence of >50 % stenosis in all three major coronary arteries, excluding left main disease.

### 2.3 Outcomes

All-cause mortality was the primary outcome of this study. Secondary outcomes included all-cause rehospitalization up to 10-year follow-up, myocardial infarction (MI), stroke, and repeat revascularization (surgical or percutaneous) up to 10 years of follow-up.

### 2.4 Statistical analysis

Summary statistics are presented as frequency (percent) for categorical variables and mean ± standard deviation (SD) for continuous variables. The primary objective of this study was to compare the long-term outcomes of PCI and CABG for patients with 3VD and HF in a major cardiac care centre in Alberta. The outcomes included all-cause death, readmission for MI (ICD codes of I21, I22, and I23 in the primary diagnosis of the readmission), readmission for stroke (ICD codes of I60, I61, I63, I64, and G45 in the primary diagnosis of the readmission), rehospitalization, and repeat revascularization at 10-year follow-up. The secondary objective was to compare the rates of referral for PCI and CABG for these patients. The index revascularization was defined as the PCI or CABG within 365 days of the angiogram. If a patient had multiple angiograms and was matched to the same revascularization, only the angiogram that was closest to the date of the revascularization would be considered as referred for the revascularization; otherwise, it would be deemed as receiving medical treatment alone. If an angiogram was linked to multiple revascularizations, only the first intervention would be treated as the index revascularization. After calculating the crude referral rate, multinomial logistic regression with PCI, CABG and medical treatment as the outcomes was used to calculate the adjusted odds ratios of receiving CABG relative to the PCI for each year, compared to year 2009, while adjusting for the covariates listed in baseline characteristics (Table 1).

**Table 1:** Baseline characteristics of patients with three-vessel disease and HF that underwent CABG or PCI.

Variables	CABG (N = 97)	PCI (N = 535)	*p* value
Age, years	65 ± 9	70 ± 12	**<0.001**
Female	24 (24.7 %)	168 (31.4 %)	0.190
Hypertension	56 (57.7 %)	386 (72.1 %)	**0.004**
Dyslipidemia	58 (59.8 %)	352 (65.8 %)	0.255
Atrial fibrillation	4 (4.1 %)	10 (1.9 %)	0.165
COPD	6 (6.2 %)	58 (10.8 %)	0.162
CEVD	6 (6.2 %)	39 (7.3 %)	0.697
Diabetes	47 (48.5 %)	221 (41.3 %)	0.190
Malignancy	2 (2.1 %)	12 (2.2 %)	0.911
CKD	0 (0 %)	11 (2.1 %)	0.154
PAD	2 (2.1 %)	42 (7.9 %)	**0.039**
Current smoker	33 (34 %)	101 (18.9 %)	**<0.001**
Former smoker	20 (20.6 %)	154 (28.8 %)	0.098
NSTEMI/UA	55 (56.7 %)	335 (56.7 %)	0.270
Stable Angina	4 (4.1 %)	25 (4.7 %)	0.812
LVEF			0.752
>50	13 (13.4 %)	62 (11.6 %)	
≥35 and ≤50	37 (38.1 %)	210 (39.3 %)	
<35	43 (44 %)	227 (42.4 %)	
Missing	4 (4.1 %)	36 (6.7 %)	
Medications			
ASA	57 (58.8 %)	359 (67.1 %)	0.111
Other antiplatelets	20 (20.6 %)	234 (43.7 %)	**<0.001**
Statin	54 (55.7 %)	261 (48.8 %)	0.212
ACEi/ARB	56 (57.7 %)	290 (54.2 %)	0.521
Beta-blocker	54 (55.7 %)	309 (57.8 %)	0.702
Anticoagulants	12 (12.4 %)	96 (17.9 %)	0.180

HF: heart failure. CABG: coronary artery bypass grafting. PCI: percutaneous coronary intervention. COPD: chronic obstructive pulmonary disease. CEVD: cerebrovascular disease. CKD: chronic kidney disease. PAD: peripheral arterial disease. NSTEMI/UA: non-ST-elevation myocardial infarction/unstable angina. LVEF: left ventricular ejection fraction. ASA: acetylsalicylic acid. ACEi/ARB: angiotensin-converting enzyme inhibitors/angiotensin receptor blockers.

Bolded p values indicate statistical significance.

Cox proportional hazards regression models and competing risk [17] were implemented to estimate the hazard ratios of the difference on the fatal and non-fatal outcomes at 10 years follow-up, with adjustment of covariates including age, sex, hypertension, dyslipidemia, atrial fibrillation, COPD, CEVD, diabetes, malignancy, CKD, PAD, current smoker, former smoker, NSTEMI/UA, stable angina, LVEF, ASA, other antiplatelets, statin, ACEi/ARB, beta-blocker, and anticoagulants (Table 2). The Kaplan-Meier curve was plotted for all-cause mortality at 10-year follow-up, and the cumulative incidence curve was also provided for all non-fatal outcomes (Fig. 3). Data extraction/analysis methods were uniform throughout follow-up.

**Table 2:** Comparison of long-term outcomes between CABG and PCI for patients with three vessel disease and HF.

Outcomes	CABG^a^ (N = 97)	PCI^a^ (N = 535)	Adjusted HR (95 % CI) (PCI as the reference)^b^	*p* value
Death up to 10 years	42 (62.4 %)	343 (71.8 %)	0.65 (0.47, 0.92)	**0.014**
Rehospitalization up to 10 years	66 (75 %)	425 (81.4 %)	0.82 (0.62, 1.08)	0.157
Readmission for MI up to 10 years	3 (3.2 %)	122 (23.7 %)	0.11 (0.03, 0.38)	**<0.001**
Readmission for stroke up to 10 years	6 (6.4 %)	37 (7.8 %)	0.87 (0.34, 2.24)	0.767
Repeat revascularization up to 10 years	6 (6.4 %)	114 (21.6 %)	0.22 (0.09, 0.53)	**<0.001**

HF: heart failure CABG: coronary artery bypass grafting. PCI: percutaneous coronary intervention. MI: myocardial infarction.

a The failure rate in the parentheses were estimates from Kaplan Meier curve or cumulative incidence curve at the longest follow up.

b Adjusting for baseline characteristics including age, sex, hypertension, dyslipidemia, atrial fibrillation, COPD, CEVD, diabetes, malignancy, CKD, PAD, current smoker, former smoker, NSTEMI/UA, stable angina, LVEF, ASA, other antiplatelets, statin, ACEi/ARB, beta-blocker, and anticoagulants.

A sensitivity analysis of time-dependent Cox models was conducted to examine the impact of immortal time bias due to the waiting period from the date of angiogram to the date of revascularization (Supplementary Table 2). Statistical analyses were executed using the SAS 9.4 (SAS Institute, Cary NC). A *p*-value < 0.05 was deemed statistically significant. All statistical tests were two-sided.

## 3 Results

Between January 1, 2009 and December 31, 2018, 1774 patients with three-vessel disease and heart failure underwent coronary angiography. We excluded patients with STEMI (n = 310), prior CABG (n = 220), those who received medical therapy only (n = 625), and those with prior revascularization before the index procedure (n = 157). A total of 632 patients underwent isolated revascularization and were included (PCI n = 535 [85 %]; CABG n = 97 [15 %]) (Fig. 1).

### 3.1 Baseline characteristics

Baseline characteristics of patients with 3VD and HF who underwent CABG or PCI are shown in Table 1. CABG patients were significantly younger (65 ± 9 vs 70 ± 11, *p* < 0.001), fewer had hypertension (57.7 % vs 72.1 % *p* = 0.004), peripheral artery disease (2.1 % vs 7.9 % *p* < 0.039), and more identified as current smokers (34 % vs 18.9 % *p* < 0.001). They were also on fewer non-ASA antiplatelets (20.6 % vs 43.7 %, *p* < 0.001). Clinical presentation (NSTEMI/UA vs stable angina) was similar between groups (*p* = 0.270, *p* = 0.812, respectively).

### 3.2 Morbidity and mortality

**Fig. 2:**
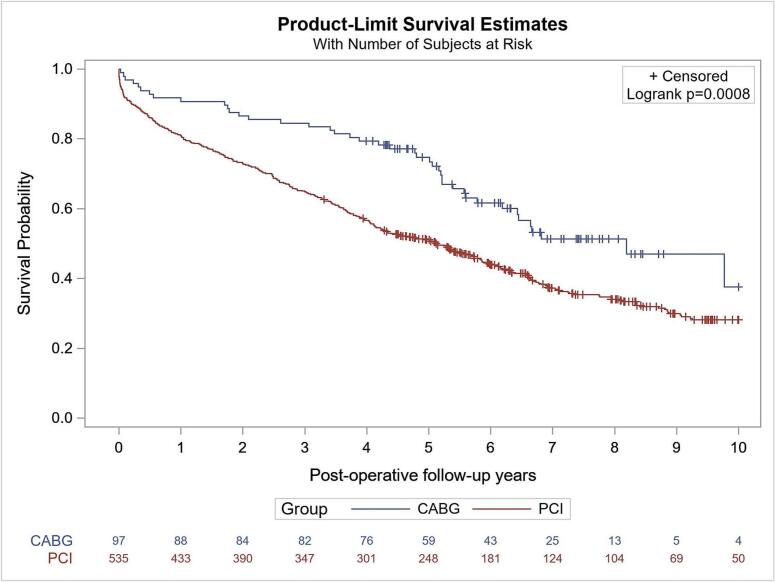
Kaplan-Meier Curve of all-cause death for 10 years follow up.

**Fig. 3:**
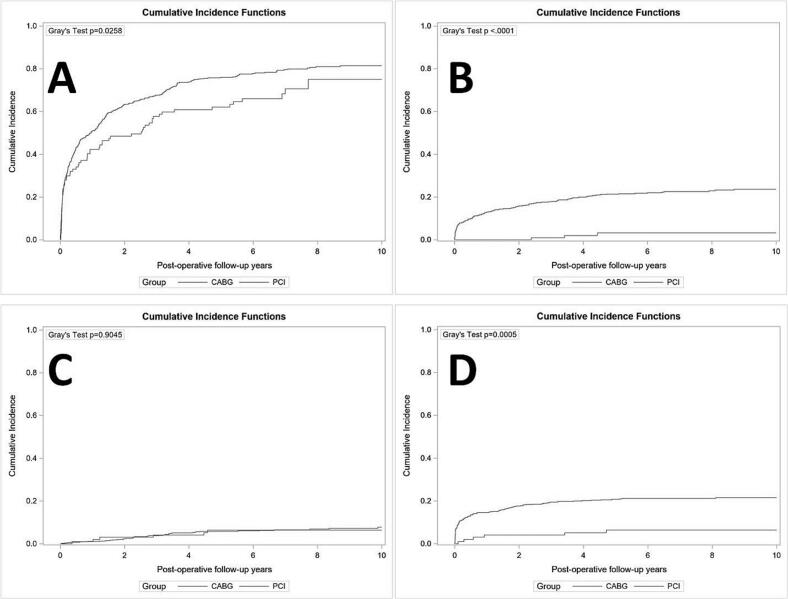
Cumulative Incidence Curve for non-fatal outcomes for 10 years of follow up. A: Readmission; B: readmission for myocardial infarction; C: readmission for stroke; D: repeat revascularization.

All-cause mortality at the 10-year follow-up was significantly lower in patients undergoing CABG (62.4 %) compared to PCI (71.8 %) (Table 2, Fig. 2) (adjusted hazard ratio (aHR) 0.65, 95 % confidence interval (CI) 0.47–0.92, *p* = 0.014). Rates of readmission for MI at 10 year follow-up were also significantly lower in patients undergoing CABG (3.2 %) versus PCI (23.7 %) (aHR 0.11, 95 % CI 0.03–0.38, *p* < 0.001) as well as rates of repeat revascularization for patients undergoing CABG (6.4 %) versus PCI (21.6 %) (aHR 0.22, 95 % CI 0.09–0.53, *p* ≤ 0.001). Rates of rehospitalization (*p* = 0.157) and readmission for stroke (*p* = 0.767) did not differ significantly between groups over the 10 year follow-up period. Kaplan-Meier estimates mirrored these results with lower rates of readmission for MI (Fig. 3B) and repeat revascularization (Fig. 3D) in patients who had undergone CABG. There was similarly no evidence on the differences in all-cause rehospitalizations (Fig. 3A) or readmissions for stroke (Fig. 3C). Sensitivity analysis of time-dependent Cox models (Supplementary Table 2) demonstrated consistent adjusted hazard ratios, indicating that our conclusions were independent of immortal time bias or survival bias.

### 3.3 Trends of CABG and PCI over the inclusion period

From 2009 to 2018, the proportion of eligible patients with 3VD and HF undergoing CABG or PCI changed significantly (Table 3). In 2009, 2.3 % of patients were referred for CABG, while 36.2 % of patients were referred for PCI, and 61.5 % were treated with medical therapy only. In 2018, 14 % of patients were referred for CABG, while 38.2 % of patients were referred for PCI, and 47.8 % were treated with medical therapy only. Overall, in the span of 9 years, patients undergoing CABG over PCI increased while the patients receiving medical treatment alone declined.

**Table 3:** Trends of CABG and PCI rates among patients with three-vessel disease and HF from 2009 to 2018 (adjusted by multinomial logistic regression).

Year	CABG (N = 97)	PCI (N = 535)	Medical treatment alone (N = 625)	Adjusted odds ratio (95 % CI) of receiving CABG over PCI⁎	*p* value
2009	3 (2.3 %)	31 (36.2 %)	80 (61.5 %)	Reference year	
2010	2 (2.1 %)	26 (42.6 %)	52 (55.3 %)	0.80 (0.12, 5.16)	0.811
2011	1 (0.9 %)	36 (39.3 %)	64 (59.8 %)	0.37 (0.04, 3.81)	0.404
2012	2 (1.5 %)	43 (42.8 %)	77 (55.8 %)	0.49 (0.08, 3.15)	0.452
2013	3 (2.4 %)	44 (44.9 %)	67 (52.8 %)	0.80 (0.15, 4.32)	0.797
2014	14 (10.2 %)	40 (41.6 %)	66 (48.2 %)	3.63 (0.93, 14.1)	0.063
2015	22 (17 %)	38 (39.2 %)	57 (43.9 %)	7.36 (1.90, 28.6)	**0.004**
2016	16 (12.6 %)	45 (47.2 %)	51 (40.2 %)	5.23 (1.33, 20.6)	**0.018**
2017	15 (11.5 %)	52 (53.4 %)	46 (35.1 %)	3.26 (0.84, 12.7)	0.089
2018	19 (14.0 %)	42 (38.2 %)	65 (47.8 %)	5.56 (1.43, 21.6)	**0.013**

⁎ Adjusting for baseline characteristics including age, sex, hypertension, dyslipidemia, atrial fibrillation, COPD, CEVD, diabetes, malignancy, CKD, PAD, current smoker, former smoker, NSTEMI/UA, stable angina, ASA, other antiplatelets, statin, ACEi/ARB, beta-blocker, and anticoagulants.

## 4 Discussion

Our retrospective study demonstrates several key findings. In patients with 3VD and a clinical diagnosis of HF, CABG was associated with better long-term outcomes at 10 years compared to PCI. Patients who underwent CABG were more likely to survive, have less readmission for MI and less repeat revascularization at 10 years. Overall, rehospitalization and readmission for stroke did not differ significantly between the groups.

Patients with HF have largely been excluded from major revascularization trials to date. Synergy Between PCI With TAXUS and Cardiac Surgery (SYNTAX) trial [10] and Future Revascularization Evaluation in Patients With Diabetes Mellitus: Optimal Management of Multivessel Disease (FREEDOM) trials [11] had less than 3 % of their study population with HF, making the study on this patient population underpowered. Moreover, the Clinical Outcomes Utilizing Revascularization and Aggressive Drug Evaluation (COURAGE) trial [12] and the International Study of Comparative Health Effectiveness With Medical and Invasive Approaches (ISCHEMIA) trial [13] did not include CAD patients with HF. Only two trials to date have assessed the efficacy of revascularization in patients with ischemic HFrEF, yet neither compares CABG and PCI directly. Surgical Treatment for Ischemic Heart Failure Extension Study (STITCHES) trial [14] compared those with ischemic cardiomyopathy to CABG or medical therapy, and Percutaneous Revascularization for Ischemic Left Ventricular Dysfunction (REVIVED-BCIS2) study [15] compared PCI to medical therapy in this patient population.

The STICHES trial found improved overall survival at 10 years compared to medical therapy (HR = 0.78, 95 % CI 0.66–0.93, *P* = 0.006) with 7 % absolute mortality reduction and fewer cardiovascular-related hospitalizations with CABG compared to medical therapy [14]. The survival of the CABG cohort in this study of 38 % at 10 years is comparable to the 41.1 % survival at 9.8-year median follow-up in STITCHES. In contrast, the REVIVED-BCIS2 study showed no benefit in terms of all-cause mortality or HF hospitalization in the PCI arm despite the short study follow-up time [15].

Several observational studies have been conducted directly comparing CABG to PCI in patients with multivessel CAD and HF, and the results favour CABG over PCI in terms of mortality, although slight differences in secondary outcomes have been reported. Retrospective registry studies done in Ontario, Australia, Sweden, and Korea have all revealed reduced long-term mortality with CABG, although morbidity outcomes differ between the studies [[18], [19], [20], [21]]. Our outcomes are consistent with these studies highlighted; however, we did not find any increase in stroke risks in patients undergoing CABG, as was seen in the Australian registry study [21]. Other secondary outcomes reported in our study, such as increased rehospitalization due to MI and increased repeat revascularization rate in the PCI group, were consistent with other observational studies [[18], [19], [20]].

Despite the evidence in support of CABG in retrospective studies, the lack of definitive large trials that compare the two revascularization methods directly leaves room for debate in this patient population group. As a result, a significant proportion of patients continue to receive PCI even in contemporary cohorts, as highlighted by the treatment trends presented in this study. This study provides long-term follow-up in a population-based cohort with 10-year outcomes and adjusted statistics demonstrating reduced rates of mortality, readmission for MI, and repeat revascularization with CABG compared to PCI. This study is further strengthened by the usage of data from a provincial database, where data from even peripheral sites within the province are recorded, which may not be available in other studies.

While the findings of this study support the use of CABG over PCI for patients with MVD and HF, treatment selection in clinical settings depends on numerous factors. Even within this patient population, there are groups that will benefit from medical therapy, PCI, or CABG. Treatment of multivessel CAD in patients with HF will depend on patient presentation, comorbidities, surgical risk, complexity of coronary artery disease, and patient preference, among other factors. As shown in the ASCERT study, which linked the STS Adult Cardiac Surgery Database to Medicare claims in 348,341 CABG patients, and found that chronic comorbidities and smoking drive late mortality. Thus, it is imperative that comprehensive clinical profiling of patients be taken into account to optimize long-term survival [22]. Medical therapy, PCI, and CABG are all essential tools in managing patients with coronary artery disease, and patients are best served by a multidisciplinary heart team review where all aspects of their medical profile may be reviewed, and the optimal individualized treatment strategy can be discussed and offered. Continued investigation into this field with an emphasis on long-term outcomes, contemporary revascularization techniques and medical treatment, as well as reductions in overall morbidity and mortality, is necessary to advance the state of the art and to continue to provide optimal treatment to all patients with coronary artery disease.

## 5 Limitations

There are several limitations to this study. First, this is a retrospective study, and patients are not randomized to treatment, and we cannot exclude residual confounding or residual bias associated with the choice for revascularization strategy. The study also used data from a single center, which may limit generalizability. Over time, clinical practices have changed, including heart failure optimization, patient selection for revascularization, the generation of stents used in PCI, and graft choices in CABG, which may influence the results presented. The exact reason for referral to the treatment arms of this study cannot be determined such as clinical choice, patient choice, or resource limitation. The study was restricted to patients undergoing revascularization; outcomes for patients treated with medical therapy alone were not analyzed. Additionally, because staged PCI could not be consistently identified, all revascularizations after the index procedure were counted as repeat revascularizations. Lastly, our analysis was limited by the absence of detailed procedural data, frailty measures, and formal surgical risk scores (e.g., STS and Euroscore), as well as detailed anatomic data and measures of coronary complexity (e.g., SYNTAX score).

## 6 Conclusions

The optimal revascularization strategy in patients presenting with three-vessel disease and heart failure remains debated due to underpowered randomized controlled trials and a limited number of observational studies with long follow-up duration. In this study, we provide a long-term follow-up of patients with heart failure undergoing CABG or PCI for three-vessel coronary artery disease. We found that CABG was significantly associated with lower long-term mortality, fewer readmissions for myocardial infarction, and fewer repeat revascularizations. Given the observational design, these findings should inform, not replace, a multidisciplinary Heart Team review to ensure the optimal intervention strategy.

## Funding list

None.

## CRediT authorship contribution statement

**Jimmy Kang:** Writing – original draft, Investigation, Data curation, Conceptualization. **Ryaan El-Andari:** Writing – original draft, Investigation, Data curation, Conceptualization. **Nicholas Fialka:** Writing – original draft, Investigation, Conceptualization. **Yongzhe Hong:** Writing – review & editing, Investigation, Formal analysis, Data curation, Conceptualization. **Michael S. McMurtry:** Writing – review & editing, Validation, Investigation, Conceptualization. **Jeevan Nagendran:** Writing – review & editing, Validation, Investigation, Conceptualization. **Jayan Nagendran:** Writing – review & editing, Supervision, Methodology, Investigation, Conceptualization.

## Ethics

This study was performed in compliance with relevant laws and institutional guidelines and have been approved by the appropriate institutional committee. Ethics approval was obtained from the University of Alberta research ethics board on November 6, 2023 for study ID Pro00124631 with individual waiver of consent.

## Declaration of competing interest

The authors declare that they have no known competing financial interests or personal relationships that could have appeared to influence the work reported in this paper.

## Data Availability

Data associated with the article is available on reasonable request to the corresponding author.

## Appendix A Supplementary data

Supplementary tables Image 1

@@@@
