# Supplementary material for: Ten-year outcomes of coronary artery bypass grafting versus percutaneous coronary intervention in patients with three-vessel disease and heart failure
Source: Am Heart J Plus. 2025 Oct 30;60:100659. doi: 10.1016/j.ahjo.2025.100659 (PMC12634857; doi:10.1016/j.ahjo.2025.100659)
Supplement: Supplementary file 1 — Supplementary tables [file mmc1.docx]

**Ten-Year Outcomes of Coronary Artery Bypass Grafting Versus Percutaneous Coronary Intervention in Patients With Three-Vessel Disease and Heart Failure**

**Supplement**

**Supplementary Table 1.** ICD codes used to define Heart Failure

| HF used ICD codes, the definition includes the following: |
| --- |
| I50.0 Congestive heart failure  I50.1 Left ventricular failure, unspecified  I50.2 Systolic (congestive) heart failure  I50.20 Unspecified systolic (congestive) heart failure  I50.21 Acute systolic (congestive) heart failure  I50.22 Chronic systolic (congestive) heart failure  I50.23 Acute on chronic systolic (congestive) heart failure  I50.3 Diastolic (congestive) heart failure  I50.30 Unspecified diastolic (congestive) heart failure  I50.31 Acute diastolic (congestive) heart failure  I50.32 Chronic diastolic (congestive) heart failure  I50.33 Acute on chronic diastolic (congestive) heart failure  I50.4 Combined systolic (congestive) and diastolic (congestive) heart failure  I50.40 Unspecified combined systolic (congestive) and diastolic (congestive) heart failure  I50.41 Acute combined systolic (congestive) and diastolic (congestive) heart failure  I50.42 Chronic combined systolic (congestive) and diastolic (congestive) heart failure  I50.43 Acute on chronic combined systolic (congestive) and diastolic (congestive) heart failure  I50.82 Biventricular heart failure  I50.83 High output heart failure  I50.84 End stage heart failure  I50.89 Other heart failure  I50.9 Heart failure, unspecified |

**Supplementary Table 2.** Sensitivity analysis time-dependent Cox model comparing long-term outcomes between CABG and PCI for patients with three vessel disease and HF

| **Outcomes** | **CABG^a^**  **(N=97)** | **PCI^a^**  **(N=535)** | **Adjusted HR(95% CI)**  **(PCI as the reference)^b^** | **P value** |
| --- | --- | --- | --- | --- |
| Death up to 10 years | 42(62.4%) | 343(71.8%) | 0.70(0.49, 0.98) | **0.037** |
| Rehospitalization up to 10 years | 66(75%) | 425(81.4%) | 0.82(0.56, 1.20) | 0.306 |
| Readmission for MI up to 10 years | 3(3.2%) | 122(23.7%) | 0.13(0.04, 0.46) | **<0.001** |
| Readmission for stroke up to 10 years | 6(6.4%) | 37(7.8%) | 0.90(0.34, 2.38) | 0.832 |
| Repeat revascularization up to 10 years | 6(6.4%) | 114(21.6%) | 0.28(0.11, 0.70) | **<0.007** |

HF: Heart failure CABG: coronary artery bypass grafting. PCI: percutaneous coronary intervention. MI: myocardial infarction.

^a^The failure rate in the parentheses were estimates from Kaplan Meier curve or cumulative incidence curve at the longest follow up

^b^ Adjusting for baseline characteristics including age, sex, hypertension, dyslipidemia, atrial fibrillation, COPD, CEVD, Diabetes, Malignancy, CKD, PAD, Current smoker, Former smoker, NSTEMI/UA, Stable Angina, LVEF, ASA, other antiplatelets, Statin, ACEi/ARB, Beta-blocker , and anticoagulants.
